# Medical Society of the County of Erie

**Published:** 1895-07

**Authors:** Franklin C. Gram

**Affiliations:** Secretary


### MEDICAL SOCIETY OF THE COUNTY OF ERIE.

Reported by FRANKLIN C. GRAM, M. D., *Secretary*.

THE semi-annual meeting of the Medical Society of the County of Erie was held in the rooms of the Buffalo Academy of Medicine, June 11, 1895, at 10 A. M., the president, Dr. FREDERICK W. BARTLETT, in the chair.

The minutes of the annual meeting were read and approved.

Dr. G. W. McPHERSON, chairman of the committee on membership, reported favorably upon the following candidates, who were thereupon unanimously elected to membership: Drs. Clara E. Bowen, Evangeline Carroll, Walter N. Kidder, Frank H. Field, Thos. B. Carpenter, Homer J. Grant, Hiram A. Kendall, Lawrence G. Hanley and P. H. Hourigan. The applications of Drs. Lounsberry, Woodruff and Milliner were laid over until the next meeting.

Applications for membership were received from Drs. Amelia F. Dresser, Jacob Miller, C. G. Fisher, Ray H. Johnson and John E. Bacon. Referred to the committee on membership.

Dr. J. B. COAKLEY, chairman of the board of censors, submitted a verbal report, in which he stated that since the last meeting two cases had been investigated by the board. The first was in relation to Dr. Ralph E. Brooks, of Marilla, against whom an anonymous complaint with no specific charges had been made, and in which case no action had been taken beyond referring the matter to the district attorney. The other was in relation to Dr. C. H. W. King, of 397 Eagle street, Buffalo, who was found to be practising medicine without being registered, as required by law, although he held a diploma from the Buffalo University. The chairman advised him in the matter, and asked the society for instructions what to do with this and similar cases.

Dr. EDWARD CLARK moved to adopt the report, and direct the board of censors to notify the district attorney of all violations of law and request him to prosecute.

Dr. C. E. CONGDON moved, in amendment, that the board of censors be authorized to draw upon the treasurer of this society for any amount required to investigate illegal practitioners and to secure the enforcement of the law.

Dr. CLARK said the district attorney is paid by the county for doing such work, and if he does not enforce the law the proper thing for this society to do is to turn its attention to the district attorney for his neglect of duty.

The amendment of Dr. Congdon was adopted.

Papers were read on Protection to the Physician, by Dr. Bartlett; Restriction and Prevention of Consumption, by Dr. B. G. Long; and Autobiographies of our Members, by Dr. Gram. In the last-mentioned paper the writer called attention to the fact that this society was incorporated in 1821, and would, therefore, soon be seventy-five years old. He suggested a proper observance of this event, and further suggested that a suitable book be secured in which could be placed biographies of deceased and autobiographies of living members, together with their portraits. In time this would become one of the most valuable books in possession of the society.

Discussion followed the reading of each paper.

On motion of Dr. Lucien Howe, the president was authorized to appoint a committee to take into consideration the proper celebration of the society's seventy-fifth anniversary.

On motion of Dr. W. C. Callanan, the secretary and librarian were instructed to send circulars to members, embodying the ideas suggested in Dr. Gram's paper, and requesting them to assist in compiling and collecting such biographies and portraits.

A representative of the Automatic Telephone Company was granted the privilege of the floor to give some information regarding his company.

As the hour for adjournment had arrived, Dr. Dunham withdrew his paper, which had been placed upon the program, and the society then adjourned.

